# Contralateral interhemispheric transfalcine approach to precuneal glioblastoma: fluorescein guided microsurgical resection and endoscopic microinspection tool

**DOI:** 10.3171/2021.10.FOCVID21195

**Published:** 2022-01-01

**Authors:** Adam M. Olszewski, Bruce I. Tranmer, Brandon D. Liebelt

**Affiliations:** Department of Neurosurgery, University of Vermont Medical Center, Burlington, Vermont

**Keywords:** interhemispheric transfalcine, endoscope assisted, fluorescein, glioblastoma

## Abstract

Maximum safe resection remains a primary goal in the treatment of glioblastoma, with gross-total resection conveying additional survival benefit. Multiple intraoperative visualization techniques have been developed to improve the extent of resection. Herein, the authors describe the use of fluorescein and endoscopic assistance with a novel microinspection device in achieving a gross-total resection of a deep seated precuneal glioblastoma. An interhemispheric transfalcine approach was utilized and microsurgical resection was completed with fluorescein guidance. A 45° endoscope was then used to inspect the resection bed, and remaining areas of concern were then resected under endoscopic visualization.

The video can be found here: https://stream.cadmore.media/r10.3171/2021.10.FOCVID21195

## Transcript

### 0:20 Patient Presentation.

This video demonstrates a contralateral interhemispheric transfalcine approach to a precuneal glioblastoma with the use of fluorescein-guided surgery and an endoscopic microinspection device. The patient is a 40-year-old man who presented to the emergency department with a 2-day history of headaches and photophobia. Past medical history was significant only for hypertension. He had a nonfocal physical exam, and a CT of his head revealed edema in the deep right parietal lobe.

### 0:53 Imaging.

His MRI is depicted here and shows a rim-enhancing lesion deep in the subcortical region in the right parietal lobe. Further workup included a CT of his chest, abdomen, and pelvis which was negative for malignancy. He then underwent a stereotactic brain biopsy which revealed the diagnosis of high-grade glioma.^1,2^ Other approach options were considered and included a right parietal transcortical or transsulcal approach with monitoring. However, given the deep and medial location in the parietal lobe, the closest pial surface was actually medially along the falx. For this reason, a left parietal interhemispheric approach was chosen to minimize transgression of the normal brain. Careful planning with preoperative imaging and neuronavigation to avoid bridging veins is imperative during this approach to prevent complications.^2,3^

### 1:44 Demonstration of Positioning.

Fluorescein dye is given intravenously just prior to induction of anesthesia upon entering the room at a dose of 3 mg/kg. The patient is positioned supine with a bump under the right shoulder and the head turned to the left. The head is elevated approximately 45° from the horizontal to allow gravity-assisted retraction of the left hemisphere in order to permit access to the deep tumor on the right side. A linear incision is used in the left parietal area just crossing the midline in order to permit exposure of the sagittal sinus.

### 2:19 Description of Craniotomy and Approach.

An oval-shaped burr hole is placed over the sagittal sinus, and a bone flap is elevated in left parietal area. A piece of Gelfoam is placed over the sagittal sinus to prevent desiccation and thrombosis. The dura is then opened in a C-shaped fashion and the dura is flapped medially over the sinus and secured with stay sutures. Interhemispheric dissection then begins. We use a Rhoton microdissector to free the adhesions along the falx and the medial surface of the parietal lobe. A large venous complex is encountered, and with persistent dissection we are able to dissect out the individual veins. After further inspection we can preserve the majority of the complex, needing to sacrifice only one of the veins to permit a wide enough corridor for tumor resection. Telfa patties are lined along the medial surface of the parietal lobe to protect the brain.

### 3:21 Opening the Falx.

We then utilize neuronavigation to plan our opening in the falx to permit access to the most superficial component of the tumor. A sharp nerve hook is then utilized to pierce the falx and pull the falx away from the contralateral hemisphere. Monopolar cautery is then transmitted through the nerve hook in order to open the falx. Tubing is placed down the axis of the nerve hook in order to prevent thermal injury to the adjacent tissues. Scissors then expand the opening in the falx, and the Telfa patties are advanced to the contralateral side. Rolled cottonoid patties can then be placed laterally in order to further widen the exposure.

### 3:55 Corticotomy.

Neuronavigation is then used to plan the cortical entry site, and bipolar cautery is utilized to start a corticotomy. This is then further opened with microscissors and deepened to expose underlying tumor.

### 4:39 Fluorescent Visualization of Tumor.

The yellow 560 filter is then utilized for fluorescent visualization of the tumor.^4,5^ The tumor is seen in yellow-green color, and this corresponds with the areas of contrast-enhancing tumor on the MRI scan. Samples are taken for pathologic analysis and an ultrasonic aspiration device is utilized to internally debulk the tumor.

### 5:07 Resection Under White Light.

The resection continues under white light, and we begin to appreciate the margin between gross tumor and adjacent normal brain on the posterior margin of the cavity. A brain ribbon retractor is used intermittently against the falx to help maximize the viewing angles for visualization of the deep-seated tumor.

### 5:32 Fluorescein-Guided Tumor Resection.

We then proceed with fluorescein-guided resection of the tumor. This fluorescent visualization is particularly useful along the margins of the contrast-enhancing portions of the tumor. In conjunction with neuronavigation, this helps to increase confidence in the discernment at the margins of the tumor cavity.

### 5:56 Description of Need for Better Visualization.

We then turn our attention to an area of a relative blind spot superiorly and laterally within the resection cavity. This is one area where it is somewhat difficult with microscopic visualization as you are trying to look around a corner with the microscope. Optimal positioning with gravity assistance does help the tumor to fall into the field; however, direct visualization at this angle is still somewhat difficult.

### 6:30 Endoscopic Inspection and Resection.

To help address this, we then bring in an endoscopic microinspection device, which is coupled with the microscope, to provide additional views and to look around corners for further tumor resection.^6,7^ This device has a 45° viewing angle, which allows us improved visualization of the areas which are not readily apparent with microscope. Final resection of residual areas of tumor in the superior and lateral quadrants of the resection cavity are performed. You can appreciate the improved visualization which is afforded by the microinspection device compared to traditional microscopic views.

### 7:18 Alternate Microinspection Device Orientation.

The orientation of the microinspection device is then rotated 180° in order to view medially as we insert the device into the resection cavity, completing our 360° panoramic inspection of the resection site.

### 7:35 Postoperative Course.

The patient did well postoperatively with no neurological deficits and was discharged home on postoperative day 2. Postoperative MRI depicted here shows an excellent resection of the tumor. There is a small focus of enhancement on the axial images, which is the location of the previous stereotactic biopsy trajectory. Diffusion-weighted imaging on the left shows no evidence of ischemia. FLAIR imaging shows expected persistent edema adjacent to the resection cavity and no new edema along the approach trajectory.

## Disclosures

The authors report no conflict of interest concerning the materials or methods used in this study or the findings specified in this publication.

## Author Contributions

Primary surgeon: Liebelt. Assistant surgeon: Olszewski, Tranmer. Editing and drafting the video and abstract: Liebelt, Olszewski. Critically revising the work: Liebelt, Olszewski. Reviewed submitted version of the work: Liebelt, Olszewski. Approved the final version of the work on behalf of all authors: Liebelt. Supervision: Liebelt.
